# Perceived Sufficiency of Full-Field Digital Mammograms With and Without Irreversible Image Data Compression for Comparison with Next-Year Mammograms

**DOI:** 10.1007/s10278-010-9277-6

**Published:** 2010-02-17

**Authors:** Stamatia Destounis, Patricia Somerville, Philip Murphy, Posy Seifert

**Affiliations:** Elizabeth Wende Breast Care, LLC, 170 Sawgrass Drive, Rochester, NY 14620 USA

**Keywords:** Digital mammography, data compression, image compression, mammogram wavelet processing, lossless compression, PACS

## Abstract

Problems associated with the large file sizes of digital mammograms have impeded the integration of digital mammography with picture archiving and communications systems. Digital mammograms irreversibly compressed by the novel wavelet Access Over Network (AON) compression algorithm were compared with lossless-compressed digital mammograms in a blinded reader study to evaluate the perceived sufficiency of irreversibly compressed images for comparison with next-year mammograms. Fifteen radiologists compared the same 100 digital mammograms in three different comparison modes: lossless-compressed vs 20:1 irreversibly compressed images (mode 1), lossless-compressed vs 40:1 irreversibly compressed images (mode 2), and 20:1 irreversibly compressed images vs 40:1 irreversibly compressed images (mode 3). Compression levels were randomly assigned between monitors. For each mode, the less compressed of the two images was correctly identified no more frequently than would occur by chance if all images were identical in compression. Perceived sufficiency for comparison with next-year mammograms was achieved by 97.37% of the lossless-compressed images and 97.37% of the 20:1 irreversibly compressed images in mode 1, 97.67% of the lossless-compressed images and 97.67% of the 40:1 irreversibly compressed images in mode 2, and 99.33% of the 20:1 irreversibly compressed images and 99.19% of the 40:1 irreversibly compressed images in mode 3. In a random-effect analysis, the irreversibly compressed images were found to be noninferior to the lossless-compressed images. Digital mammograms irreversibly compressed by the wavelet AON compression algorithm were as frequently judged sufficient for comparison with next-year mammograms as lossless-compressed digital mammograms.

To date, adoption of full-field digital mammography (FFDM), especially in the USA, has been slowed by financial and workflow issues.^1^,^2^ Integration of FFDM systems into preexisting picture archiving and communications systems (PACS)—for the purposes of achieving the workplace efficiencies that can derive from digitized transmission, storage, and manipulation of images—has been difficult. File sizes associated with digital mammographic images after lossless compression are extremely large.^3^–^6^ Depending on the type of FFDM system used, individual lossless-compressed digital mammograms range in size from 2.5 to 16 MB. With four views required during a standard screening examination, patient files can thus routinely approach 60 MB, depending on the FFDM detector.^7^ With many current PACS, the transmission, retrieval, and processing of such large image sizes and patient files can impair overall network speed. Costs for an adequate PACS storage capacity are decreasing over time as the technology matures, but they remain relatively high.

The concept of irreversible (or lossy) image compression for digital mammograms has been met with skepticism, particularly in the USA, because of concerns about image degradation,^7^ but irreversible image compression might be desirable if, in combination with a PACS storage strategy, it can readily help realize cost and time efficiencies associated with the transmission, storage, and display of digital mammograms.

The wavelet Access Over Networks (AON) compression algorithm (FUJIFILM Medical Systems USA, Inc., Stamford, CT) was developed to enable simultaneous PACS server storage of three different versions of the same image: the “original” lossless-compressed digital mammogram (estimated file size, 2.5 to 16 MB), the digital mammogram irreversibly compressed by means of a wavelet bit-by-bit compressor at a ratio of ≤20:1 (estimated file size, 0.5 to 1.7 MB), and the digital mammogram irreversibly compressed by the means of the same compressor at a ratio >20:1 (estimated file size, 0.2 to 1.3 MB; the exact irreversible compression ratios used for PACS storage would depend on several factors in the image, such as pixel matrix and bit depth). If irreversibly compressed images are of a sufficient quality to allow their use for future clinical comparisons, the lossless digital mammograms can be archived (i.e., removed from the PACS server) after 2 to 5 weeks of storage rather than after 1 to 2 years, the typical storage time for original images of other modalities. The irreversibly compressed images could be stored for clinical comparisons on the PACS server for 3 to 5 years before archiving, and higher ratio irreversibly compressed images could be stored for reference on the PACS server for one further year before archiving.

To determine whether irreversible compression of digital mammograms at ratios ≤20:1 and >20:1 by the wavelet AON compression algorithm will yield acceptable image quality, we undertook a blinded reader study with experienced radiologists to evaluate irreversibly compressed images compared with lossless-compressed images in terms of their perceived sufficiency for comparison with next-year mammograms.

## Materials and Methods

### Study Protocol

This study was conducted at a dedicated breast screening center at which 160 to 180 FFDM examinations are performed per day and more than 80,000 screening mammographies take place per year. To participate in the study, radiologists were required to have read a minimum of 2,000 mammograms per year for each of the past 3 years or to have recently completed a 1-year fellowship in which they read at least 10,000 mammograms. Because 12 radiologists were required for the study, 16 radiologists associated were recruited, and they signed a reader-informed consent agreement. The study protocol was approved by the Western Institutional Review Board in Olympia, Washington, and the study was conducted in accordance with the ethical principles of the Declaration of Helsinki.

For a brief orientation preceding the study session, eight sample cases (comprised of cancer and noncancer patients) were chosen from images not included in this investigation. Orientation included a review of the study case report forms (CRFs) and a demonstration of the PACS workstation (Synapse, FUJIFILM Medical Systems USA, Inc) on which the study was conducted.

In the study, the reading of digital mammograms took place in a totally darkened room dedicated to reading mammograms. To maintain confidentiality, patient identifiers on each digital image were limited to a unique study number and the subject’s initials. For images containing lesions, radiologists were not told either the type of lesion or the relative position. The order in which each reader evaluated the mammography images was staggered.

Image pairs were displayed on two high-resolution (5-megapixel) grayscale portrait monitors (Coronis 5MP Mammo, BarcoView LLC, Duluth, Georgia) calibrated to the Digital Imaging and Communications in Medicine (DICOM) Part 14 Display Standard. Luminance was set at 600 cd/m^2^. Adjustments of the width and level of the image display window were not permitted. The display window width and center were set at 1,024/511, respectively. On-screen magnification up to one time was permitted (where 1 pixel displayed was equal to 1 pixel acquired). To ensure consistency and accuracy, this level of magnification was enabled by means of a hotkey on the PACS workstation.

### Description of Wavelet Compression and Selection of Study Compression Ratios

The AON compression algorithm consists of a patented set of software tools for image compression, storage, and delivery. After a digital image is acquired, the AON compression engine, residing on the PACS server, will compress the image at multiple ratios depending on intended use and network capabilities, creating up to three versions of the acquired image. The compression ratios are configurable by image type, modality manufacturer, body part, and facility preferences. Images can be lossless-compressed or irreversibly (lossy) compressed at ratios up to 100:1. The AON compression engine writes a lossless-compressed DICOM Part 10 image version as the “original” image in order to meet the guidelines of the US Mammography Quality Standards Act (MQSA). The PACS user can then select which version of the image is to be delivered to the user workstation, the choice of which version depending on clinical requirements, available bandwidth, and other factors. When a compressed image is requested by a PACS user, the file is quickly delivered, and display software on the PACS workstation decompresses the image for viewing.

For the current study, two irreversible compression values were chosen after laboratory testing was conducted using the American College of Radiography (ACR) mammography accreditation phantom (MAP) to identify maximum data compression ratios meeting the minimum scoring criteria for phantoms as defined by the MQSA. Four different irreversible compression ratios (10:1, 20:1, 40:1, and 50:1) were tested in order to select a compression ratio at ≤20:1 and another compression ratio at >20:1 for the study. A certified radiographic technologist made two exposures of the MAP, one with automatic exposure control technique and a second with the mAs readout reduced to one-third. The images were sent to a PACS workstation as lossless-compressed images and as irreversibly compressed images in the four different compression ratios. Pairs of the phantom images were then analyzed by the investigator on two 5-megapixel DOME monitors by the standard procedure of counting the number of visible objects (e.g., fibers, masses, and microcalcifications). The irreversible compression ratios of 10:1, 20:1, and 40:1 met MQSA minimum scoring criteria as judged by the investigator; the irreversible compression ratio of 50:1 did not. For this study, the irreversible compression ratios of 20:1 and 40:1 were selected as representative of the PACS storage strategy for which the AON compression algorithm was created.

### Image Database

A total of 100 breast cancer screening or diagnostic cases were selected from an existing library of digital mammograms acquired by means of a Fujifilm Digital Mammography System (FUJIFILM Medical Systems USA) either at the University of California at Los Angeles, Los Angeles, CA, or at the Mayo Clinic, Rochester, MN. The cases were acquired at these facilities under patient-informed consent, and they were used previously by FUJIFILM Medical Systems USA, Inc, for submission of a premarketing approval application. The cases were drawn from patients with known true clinical status and with complete screen-film and digital mammography examinations (four standard views), in which there was sufficient anatomical coverage, sufficient contrast, no significant motion or other artifacts, no over- or underexposure of film, limited noise, and clinically insignificant difference in subject positioning between screen-film and digital mammography as determined by the image acquisition sites and the adjudicator. The 100 cases were chosen to include 50 tissue-proven cancers and 50 noncancers, with the diagnoses of noncancers determined by tissue sampling, mammography special views, ultrasound, or 1-year follow-up. Of the 100 cases, 17 were negative with 1-year follow-up. The 100 cases were also chosen to include 50 mediolateral oblique views and 50 craniocaudal views (Table 1). A further selection criterion was that the images represent a distribution of cancers and noncancers from (1) both screening and diagnostic populations, (2) all ACR Breast Imaging Reporting and Database System (BI-RADS) categories, (3) various types of main findings, and (4) all breast tissue composition categories, including cases with heterogeneously dense or extremely dense breast tissue (Table 1).

**Table 1:** Demographic and Clinical Characteristics of Digital Mammograms

Characteristic	*N* = 100
Age (year)	58.0 ± 12.3
Caucasian race (%)	85
Breast tissue composition (%)	
Almost entirely fat (0–25%)	19
Scattered fibroglandular densities (26–50%)	30
Heterogeneously dense (51–75%)	44
Extremely dense (76–100%)	7
Image acquisition site (%)	
Mayo Clinic	67
University of California at Los Angeles	33
Population (%)	
Screening	49
Diagnostic	51
BI-RADS category (%)^a^	
1	9
2	6
3	10
4	48
5	27
True clinical status (%)	
Cancer^b^	50
Noncancer	50
One main feature (%)	
Calcification only	22
Mass only	39
Mass with calcifications	14
Architectural distortion	7
Focal asymmetry	12
No identifiable finding	6

*BI-RADS* Breast Imaging Reporting and Database System

^a^Final BI-RADS assessment category was based on the official screen-film mammography report after consideration of special views and ultrasound, if acquired

^b^Of the cancers, 42% was from a screening population, and 58% of cancers were from a diagnostic population. Of the cancers, 50% was BI-RADS category 4, and 50% were BI-RADS category 5

Study images were shown in pairs, with one image displayed per monitor, in three comparison modes: lossless-compressed images vs 20:1 irreversibly compressed images (mode 1), lossless-compressed images vs 40:1 irreversibly compressed images (mode 2), and 20:1 irreversibly compressed images vs 40:1 irreversibly compressed images (mode 3). Each reader rated all images in all three modes independently of the other readers. To ensure intrareader reproducibility, a subset of 20 cases were read twice in each of the three modes. Overall, then, each reader performed 120 reviews per comparison mode (Table 2). Compression levels were randomly assigned between the two monitors, and the order of presentation of image pairs was randomly assigned to counterbalance the order of presentation across modes (Table 3).

**Table 2:** Distribution of Image Types by Breast, View, and Cancer Status

Breast, view, and status	Reliability subset (read twice) *N* = 20	Reader review *N* = 80
RCC cancer	4	8
RCC noncancer	3	12
RMLO cancer	1	11
RMLO noncancer	3	10
LCC cancer	2	11
LCC noncancer	1	9
LMLO cancer	2	11
LMLO noncancer	4	8

*RCC* right craniocaudal, *RMLO* right mediolateral oblique, *LCC* left craniocaudal, *LMLO* left mediolateral oblique

**Table 3:** Order Sequence of Comparison Images for Display

Breast and mode	Left monitor	Right monitor
RCC or RMLO		
Mode 1	20:1 irreversibly compressed	Lossless-compressed
Mode 2	Lossless-compressed	40:1 irreversibly compressed
Mode 3	40:1 irreversibly compressed	20:1 irreversibly compressed
LCC or LMLO		
Mode 1	Lossless-compressed	20:1 irreversibly compressed
Mode 2	40:1 irreversibly compressed	Lossless-compressed
Mode 3	20:1 irreversibly compressed	40:1 irreversibly compressed

*RCC* right craniocaudal, *RMLO* right mediolateral oblique, *LCC* left craniocaudal, *LMLO* left mediolateral oblique

### Study End Points

Readers were asked to record on the CRFs whether the quality of each right-side monitor image and left-side monitor image was sufficient for comparison with next-year mammograms and which image (right or left) had the lower level of data compression. In addition, if both images were perceived as sufficient for comparison with next-year mammograms, the readers were asked about their preference between the images on a 7-point Likert scale ranging from +3 (image on the right is much better) to 0 (similar) to –3 (image on the left is much better). Secondary end points similarly evaluated on a 7-point Likert scale were which image (right or left) better displayed an object if there was an area of interest on the image, which image (right or left) better displayed the area at or near the skin line, and which image (right or left) better displayed the area at or near the chest wall.

### Statistical Analysis

A total of 12 radiologists was considered adequate for statistical analysis. Because of potential for scheduling conflicts with the time of the study, 16 radiologists were recruited to participate, and all were able to do so. For one of the 16 radiologists, however, study image pairs were accidentally flipped on the monitors in mode 1 after software at the radiologist’s workstation was restarted when the initial sequences of image pairs did not load properly. After the software was restarted, images appeared on the two monitors in the software’s default setting, not the programmed study setting. The restarting of the workstation’s software was noted by the study monitors, and the setup anomaly was afterward verified. Because of the accidental software error, data from this radiologist were excluded from the study. Data from the other 15 radiologists were used for analysis.

The primary objective of the study was to demonstrate the noninferiority of irreversibly compressed images vs lossless-compressed images in terms of perceived sufficiency of image quality for comparison with next-year mammograms. A random-effect analysis was applied to ascertain the difference between compression level pairs in each mode in terms of the per-reader average proportion of images perceived to be of sufficient quality for comparison with next-year mammograms. The Obuchowski model was used to calculate sample sizes and to compute the standard error of the per-reader average for each compression level in each mode.^8^,^9^ The Student’s *t* distribution was used to obtain confidence intervals (CIs). Although the Obuchowski model is most commonly applied to the area under the receiver operating characteristic (ROC) curve, it can by extension be applied to proportions of successes. The end point of perceived sufficiency of image quality for comparison with next-year mammograms, it should be noted, is not the same as an end point of diagnostic accuracy, for which an ROC study would have been appropriate. The irreversibly compressed images in this study are not intended for detecting or diagnosing malignancies but for making decisions about next-year mammograms.

The random-effect analysis permitted generalization of perceived sufficiency beyond the number of cases and readers in this study. Within each mode, noninferiority was considered demonstrated if the lower limit of the 98.33% CI for the average difference between levels of compression in terms of the per-reader average proportion of images perceived to be of sufficient quality exceeded the value of −0.05. The noninferiority delta was set at 5%. At an alpha level of 0.05/3, a target sample size of 100 comparison cases with 12 readers was calculated.

For overall preferences between the paired images and for preferences in terms of object display and display of areas at or near the skin line and at or near the chest wall, the alpha level was set at 0.5/3 to protect against multiple comparisons. For each comparison mode, the per-reader average proportion of scores that were at least 0 (indicating that the more compressed image has image quality similar to or better than the less compressed image) was calculated, along with corresponding 98.33% CIs.

For reader ranking of image compression, the alpha level was set at 0.05/3 to protect against multiple comparisons. For each mode, the average proportion of correct responses was calculated along with corresponding 98.33% CIs. A 98.33% CI was computed using a normal approximation and a standard error adjusted for correlations among the proportions. If the CI was <0.50, it was concluded that the images were comparable, since readers correctly identified more compressed images less often than would be expected by chance.

Intrareader reproducibility was also examined for all end points using data from the 20 cases in the subset. For each reader, in each mode, proportions of observed agreement and of expected agreement were computed. Kappa statistics were computed when the proportion of expected agreement was less than perfect.

Readers dictated responses to transcribers, who filled in supplied CRFs by pen. The CRFs were managed by PharmaPro Corporation, Inc. (Cambridge, MA). Data analysis was performed by Biostatistics Consulting, LLC (Toronto, Ontario, Canada).

## Results

Of a total expected 5,400 executed CRFs (3 × 120 image comparisons × 15 readers), 5,356 were obtained (99.2%). Data on the CRFs were very complete, with only 19 values (0.04%) missing out of a potential 48,204 values. All CRFs were evaluated as planned.

In each mode, the per-reader average proportion of images judged to be of sufficient quality for comparison with next-year mammograms at each compression level was ≥97.37% (Table 4). In mode 1, the average proportions were 97.37% for both the lossless-compressed images and the 20:1 irreversibly compressed images. In mode 2, the average proportions were 97.67% for both the lossless-compressed images and the 40:1 irreversibly compressed images. In mode 3, the average proportions were 99.33% for the 20:1 irreversibly compressed images vs 99.19% for the 40:1 irreversibly compressed images. For each of these average proportions, the 98.33% CI was ≥91.37%.

**Table 4:** Average Proportion of Images Perceived to be of Sufficient Quality for Comparison with Next-Year Mammograms

Comparison mode	Relative compression level	Perceived sufficient for comparison with next-year mammograms
1. Lossless-compressed vs 20:1 irreversibly compressed	Less compressed	97.37% (98.33% CI, 91.37–100%^a^)
	More compressed	97.37% (98.33% CI, 91.37–100%^a^)
2. Lossless-compressed vs 40:1 irreversibly compressed	Less compressed	97.67% (98.33% CI, 92.41–100%^a^)
	More compressed	97.67% (98.33% CI, 92.41–100%^a^)
3. 20:1 irreversibly compressed vs 40:1 irreversibly compressed	Less compressed	99.33% (98.33% CI, 98.12–100%^a^)
	More compressed	99.19% (98.33% CI, 97.92–100%^a^)

*CI* confidence interval

^a^The upper limit of the confidence interval was truncated at the boundary value of 100%

In the random-effect analysis that was conducted to determine the noninferiority of the more compressed images in each comparison mode, the lower limit of the 98.33% CI for the difference between compression levels in the average proportion of images perceived to be of sufficient quality for comparison with next-year mammograms was −0.23% in mode 2 and −0.35% in mode 3 (Table 5). Both lower limits lie above the a priori noninferiority threshold of −5.00%, thus confirming the statistical noninferiority of 40:1 irreversibly compressed images to both lossless-compressed images and 20:1 irreversibly compressed images. Such a CI for this difference could not be constructed in mode 1 (lossless-compressed images vs 20:1 irreversibly compressed images) because the rating of perceived sufficiency was always the same for the paired images, giving an estimated difference of 0.00% with no variance.

**Table 5:** Random-Effect Analysis of Differences in Average Proportions

Comparison mode	Difference in average proportions	SE	Lower limit of 98.33% confidence interval
1. Lossless-compressed vs 20:1 irreversibly compressed	0.00%	0.00%^a^	0.00%
2. Lossless-compressed vs 40:1 irreversibly compressed	0.00%	0.10%	−0.23%
3. 20:1 irreversibly compressed vs 40:1 irreversibly compressed	0.13%	0.09%	−0.35%

*SE* standard error

^a^The standard error is 0.00% because for each reader, whenever one image was perceived to be of sufficient quality for comparison with next-year mammograms, the other image was also perceived to be of sufficient quality for comparison with next-year mammograms, that is, there was no discordance in the ratings

When readers judged both images to be of sufficient quality for comparison with next-year mammograms, they used a category other than “similar” in <2% of the cases to express a preference between the images. When a preference was expressed, the average proportion of cases in which the more compressed image was preferred was 90.93% (98.33% CI, 83.57–98.29%) in mode 1, 89.96% (98.33% CI, 79.82–100% (truncated)) in mode 2, and 92.30% (98.33% CI, 85.40–99.21%) in mode 3.

Overall, readers were not able to identify correctly which images were less compressed (Table 6). The average proportion of image pairings in which the less compressed image was correctly identified was 17.31% for mode 1 (98.33% CI, 4.32–30.31%), 18.01% for mode 2 (98.33% CI, 5.66–30.36%), and 16.22% for mode 3 (98.33% CI, 5.20–27.24%). Because these CI values were all <50%, the images were deemed comparable because readers correctly identified fewer compressed images than would be expected by chance.

**Table 6:** Average Proportion of Image Pairings with Less Compressed Image Correctly Identified

Comparison mode	Less compressed image was correctly identified
1. Lossless-compressed vs 20:1 irreversibly compressed	17.31% (98.33% CI, 4.32–30.31%)
2. Lossless-compressed vs 40:1 irreversibly compressed	18.01% (98.33% CI, 5.66–30.36%)
3. 20:1 irreversibly compressed vs 40:1 irreversibly compressed	16.22% (98.33% CI, 5.20–27.24%)

*CI* confidence interval

Readers indicated that there was an area of interest in 70.80% of the image pairings in mode 1, 71.62% in mode 2, and 72.36% in comparison mode 3. In these cases, the more compressed image was rated as at least similar in 93.91% of the image pairings in mode 1, in 90.49% of the image pairings in mode 2, and in 93.46% of the image pairings in mode 3 (Table 7). Readers indicated that the chest wall was visible in 60.77% of the images in mode 1, 60.99% of the images in mode 2, and 60.83% of the images in mode 3. In those cases, the more compressed image was rated as at least similar in 98.38% of the image pairings in mode 1, 97.49% of the image pairings in mode 2, and 97.71% of the image pairings in mode 3. Readers indicated that the area at or near the skin line was displayed at least as well by the more compressed image as by the less compressed image in 98.44% of the image pairings in mode 1, 95.50% in mode 2, and 95.85% in mode 3.

**Table 7:** Perceived Relative Quality of More Compressed Images

Characteristic	Per-reader average proportion of image pairings in which the more compressed image was rated as at least similar
Display of object in identified area of interest	
1. Lossless vs 20:1	93.91% (98.33% CI, 86.94–100.0%^a^)
2. Lossless vs 40:1	90.49% (98.33% CI, 81.79–99.20%)
3. 20:1 vs 40:1	93.46% (98.33% CI, 86.03–100.0%^a^)
Display of the area at or near the skin line	
1. Lossless vs 20:1	98.44% (98.33% CI, 96.45–100.0%^a^)
2. Lossless vs 40:1	95.50% (98.33% CI, 90.24–100.0%^a^)
3. 20:1 vs 40:1	95.85% (98.33% CI, 90.07–100.0%^a^)
Display of the area at or near the chest wall	
1. Lossless vs 20:1	98.38% (98.33% CI, 96.45–100.0%^a^)
2. Lossless vs 40:1	97.49% (98.33% CI, 93.39–100.0%^a^)
3. 20:1 vs 40:1	97.71% (98.33% CI, 94.09–100.0%^a^)

*CI* confidence interval

^a^The upper limit of the confidence interval was truncated at the boundary value of 100%

In the intrareader reproducibility subset of 20 comparison images, a large number of readers had perfect observed and perfect expected agreement. Table 8 summarizes, for each comparison mode, the percentages of readers for whom perfect observed and expected agreement occurred in terms of the perceived sufficiency of images for comparison with next-year mammograms.

**Table 8:** Intrareader Reliability for Perceived Sufficiency of Images

Comparison mode	κ not defined *p*_o_ = *p*_e_ = 1	κ = 1 *p*_o_ = 1, *p*_e_ < 1	κ < 1
1. Lossless vs 20:1			
More compressed	86.67%	6.67%	6.67%
Less compressed	86.67%	6.67%	6.67%
2. Lossless vs 40:1			
More compressed	86.67%	0.0%	13.33%
Less compressed	86.67%	0.0%	13.33%
3. 20:1 vs 40:1			
More compressed	86.67%	6.67%	6.67%
Less compressed	86.67%	6.67%	6.67%

*p*_o_ = observed proportion of agreement, *p*_e_ = expected proportion of agreement

## Discussion

The AON irreversible compression algorithm was developed to facilitate the integration of FFDM systems into PACS. In this reader study of irreversibly compressed images compared with lossless-compressed images, the perception of sufficiency for comparison with next-year mammograms was achieved by 97.37% of the lossless-compressed images and 97.37% of the 20:1 irreversibly compressed images in mode 1, 97.67% of the lossless-compressed images and 97.67% of the 40:1 irreversibly compressed images in mode 2, and 99.33% of the 20:1 irreversibly compressed images and 99.19% of the 40:1 irreversibly compressed images in mode 3. In the random-effect analysis that was conducted in order to determine the noninferiority of the irreversibly compressed images vs lossless-compressed images in terms of perceived sufficiency for comparison with next-year mammograms, both 20:1 and 40:1 irreversibly compressed images were found to be statistically noninferior.

Although all the images in this study were derived from a single FFDM system (FUJIFILM Medical Systems), only three of the 16 readers originally recruited to the study had any previous experience with FUJIFILM digital mammography. It seems unlikely that any previous experience with digital mammograms from this source would have affected results.

Given the advantages of image compression for the transmission and storage of digital mammography files, there are very few studies that evaluate the compression ratios of mammograms.^3^–^5^ In a reader study with five experienced radiologists, Penedo and colleagues irreversibly compressed mammograms at 40:1 and 80:1 ratios using both the JPEG 2000 and object-based SPHIT methods.^4^ Readers were asked to locate and rate clusters of microcalcifications and masses on 112 original and compressed images. They found that irreversible compression ratios up to 80:1 did not decrease the rate of detection of clusters of microcalcifications and masses for either compression method. The images used in this study were digitized, not digital, mammograms.

Suryanarayanan and colleagues investigated the effect of the JPEG 2000 compression algorithm on the detection of simulated masses of various sizes and clustered microcalcifications on 100 FFDM images obtained for use in clinical practice.^5^ The compression ratios were 1:1, 15:1, and 30:1. The images were presented to five experienced readers who evaluated them on a 6-point scale. The investigators found that the JPEG 2000 compression algorithm affected the detection of microcalcifications at compression ratios >15:1.

The advantages of high compression values for the transmission and storage of digital mammography images are obvious.^10^ More than any other imaging modality, digital mammography poses difficult questions about the management of digital images. A screening and diagnostic facility that sees up to 180 patients per day could produce up to 14 GB per day. Moreover, mammography is the single area of radiology for which comparisons of current studies with past examinations are legally required. Consequently, if an average of three comparison examinations exists for every patient, the potential need for daily storage could then increase up to 42 GB per day (3 × 14 GB).

This current comparison of irreversibly compressed images with lossless-compressed images is the largest to date in terms of readers, and it focuses on the most basic and practical need: the perceived sufficiency of the images for comparison with next-year mammograms. Those digital mammograms irreversibly compressed by the AON compression algorithm at ratios of 20:1 and 40:1 were statistically noninferior to lossless-compressed digital mammograms, suggesting the feasibility of the previously outlined staged storage strategy for integrating FFDM images with PACS networks.

## Study Limitations

This comparative analysis of full-field digital mammograms with and without irreversible image data compression did not study the diagnostic accuracy of irreversibly compressed images, nor did we analyze the application of the AON compression algorithm to digital mammograms from FFDM platforms other than that of FUJIFILM Medical Systems. Our analysis did confirm that there might be value in future evaluation of the AON compression algorithm for digital mammography within the context of a PACS storage strategy.
